# Postoperative functional status in patients with supratentorial superficial low-grade glioma

**DOI:** 10.1186/s12957-017-1237-x

**Published:** 2017-10-17

**Authors:** Ji Zhang, Yin sheng Chen, You-ping Li, Zheng-quan Zhu, Jian-min Liu, Cheng-cheng Guo, Qun-ying Yang, Xiao-li Wang, Ying-hua Rao, Qing Mao, Wen-yan Li, Lu Ma, Yun qiang Yang, Shi-yin Xiao

**Affiliations:** 1 Department of Neurosurgery, State Key Laboratory of Oncology in South China, Sun Yat-sen University Cancer Center, Collaborative Innovation Center for Cancer Medicine, 651 Dong Feng East Road, Guangzhou, 510060 China; 2 Department of Neurosurgery, The First Affiliated Hospital of Nanchang University, Nanchang, Jiangxi China; 3 Department of Neurosurgery, Tumor Hospital Affiliated of Xinjiang Medical University, Xinshi District, Ürümqi, Xinjiang China; 4 Department of Neurosurgery, The First Affiliated Hospital of Guangzhou University of Traditional Chinese Medicine, Guangzhou, China; 5 Department of General Surgery, Shang Jin Nan Fu Hospital, West China Hospital, Sichuan University, Cheng du, Sichuang China; 6 Department of Neurosurgery, Guangzhou Baiyun District People’s Hospital, Guangzhou, China; 7 Department of General Surgery, West China Hospital of Sichuan University, Cheng du, Sichuang China; 8 Department of Neurosurgery, Affiliated Hospital of Guiyang Medical College, Guiyang, Guizhou Province China; 9 Department of Stomatology, Tongji Hospital, Tongji Medical College, Huazhong University of Science and Technology, Wuhan, China; 10 Department of Neurosurgery, The second Affiliated Hospital of Guangzhou Medical University, Guangzhou, China

**Keywords:** Functional status, ASS-LGG, Surgical therapy, Relative factor

## Abstract

**Background:**

We investigated the functional status of adult supratentorial superficial low-grade glioma (ASS-LGG) after surgery and analyzed its relevant factors to guide the therapeutic strategy and improve the life quality of these patients.

**Methods:**

Clinical materials from January 2008 to December 2010 in 104 adults with ASS-LGG were analyzed retrospectively. The follow-up period ranged from 6 months to 1.5 years. The logistic regression was used to evaluate the preoperative and postoperative variation of functional status in patients to disclose the relevant factors affecting postoperative functional status, such as age, gender, the duration of symptom, size and location of the tumor, hemisphere, resection degree, and tumor pathologic grade and preoperative Karnofsky performance status (Pre-KPS).

**Results:**

Four out of nine candidate factors are related to the postoperative functional status. They are age less than 40 years, the size of tumor less than 5 cm in diameter, tumor located in the right hemisphere, and limited resection of tumor in the eloquent area.

**Conclusions:**

It seems more meaningful to evaluate the functional status of the patients with ASS-LGG on the basis of these clinical features, involving age, tumor size, location, and extent of resection.

## Background

Low-grade glioma (LGG) accounts for more than 15% of adult gliomas and 25% of child gliomas [1]. The principle of surgery for LGG is to maximally protect the brain function and resect the tumor as much as possible [2, 3], but it seems difficult to reach a balance between resection degree and function protection because of no clear boundary between tumor and normal brain tissue. Thus, radical surgery versus functional protection is still a matter of debate in LGG patients [1, 3]. The Karnofsky performance status (KPS), which is usually used for making clinical decisions, measures the variation in patient performance on three domains: activity level, ability to work, and ability of self-care. Evaluating the patient’s ability to perform ordinary activities is rated on 11 levels. The total score ranged from 0 = Dead to 100 = Normal [4]. LGG progresses slowly, with the average survival period of 6 to 8 years. There are a lot of controversies about prognostic factors including age, pre-operative clinical manifestation, location of tumor, volume of tumor, and extent of resection in LGG. These studies did not explore the functional status of patients preoperatively and postoperatively. The goal of this study is to investigate the postoperative functional status variation of adult supratentorial superficial low-grade glioma (ASS-LGG) to provide the surgical strategy.

## Methods

We collect the data of patients who underwent surgery and were pathologically diagnosed as LGG in our department from Jan. 2008 to Dec. 2010. The uniform table was used to collect data including pre- and postoperative KPS, age, gender, clinical manifestation, size and location of the tumor, the duration of symptom, degree of resection, and pathologic grade. The non-conditional logistic regression was used to assess the change of functional status and analyze the factors affecting functional status. KPS was implied to elevate the functional status of patients. The alteration of KPS beyond 10 points was considered as being valuable. SPSS software package version 18.0 (SPSS Inc., Chicago, IL) was implied in statistics.

## Results

Of 112 adult patients proven with LGG surgically, 8 patients withdraw. In the rest 104 patients, the gender ratio (male/female) is 1:1.7 (38/66). The average age was 40 years old. The duration of symptom ranged from 1 week to 3 years (6 months in average). The average diameter of tumor was 5 cm. The locations of tumor were frontal lobe (31%), temple lobe (36%), parietal lobe (12%), occipital lobe (9%), and multi-lobes (12%). Fifty-seven patients’ tumors were situated in the left cerebral hemisphere, 47 in the right side. The degree of resection depended on whether the tumor was seated in the functional area or not. The surgical principle was executed as follows: tumor located in the functional area—partial resection (13%); near the functional area—subtotal resection (29%); and the non-functional area—total resection (58%). All patients acquired pathologic diagnosis: 44 (42%) were grade I and 60 (58%) were grade II. About 33% patients had combined radiological treatment following surgery.

The average KPS before surgery was 65. We followed up all patients for 6 to 18 months; two patients died from complication (KPS = 0). The mean KPS after surgery was 75. We found that four factors were associated with the postoperative functional status of patients (Table 1). They are age less than 40 years, the size of tumor less than 5 cm in diameter, tumor in the right hemisphere, and limited resection of tumor in the functional area. To patients over 40 years, their improvement of KPS was worse than the younger. Patients with the tumor’s diameter less than 5 cm have better improvement of KPS than those more than 5 cm. These patients in the right hemisphere LGG have better improvement after surgery. According to whether the tumor is located in the functional area, the patients’ improvement of KPS shows a trend as follows: partial resection (functional area) > subtotal resection (near functional area) > total resection (non-functional area).

**Table 1:** The relativity between the functional status and the relevant factors

Factors	*B*	SE	*χ* ^2^	*p*	*OR*
Gender	− 0.183	0.166	1.213	0.271	0.833
Age	− 0.086	0.340	4.064	**0.048**	0.915
Duration of symptom	0.254	0.288	0.776	0.378	1.038
Location	− 0.242	0.310	0.607	0.436	0.624
	0.278	0.621	0.200	0.655	3.105
	− 0.227	0.573	0.157	0.692	1.850
	0.841	0.379	3.908	0.127	2.319
Size	0.540	0.349	2.390	**0.038**	1.716
Hemisphere	− 0.224	0.304	4.542	**0.020**	0.799
Resection degree	− 0.410	0.208	5.885	**0.035**	0.664
	− 2.334	0.504	21.471	**0.000**	0.097
Pathologic grade	− 0.820	0.608	41.345	0.100	0.441
Pre-KPS	0.278	0.621	0.200	0.655	3.105

Gender: (1) female; (2) male. Age: (1) < 40; (2) > 40. Duration of symptom: (1) < 6 months; (2) > 6 months. Location: (0000) frontal lobe; (1000) parietal lobe; (0100) temple lobe; (0010) occipital lobe; (0001) multi-lobes. Size: (1) < 5 cm; (2) > 5 cm. Hemisphere: (1) left; (2) right. Resection: (00) total resection; (10) subtotal resection; (01) partial resection. Tumor pathologic grade: (1) WHO grade I; (2) WHO grade II. KPS: (1) ≥ 70; (2) < 70

## Discussion

For ASS-LGG, there is still controversy on the appropriate management and impact factors for functional status, such as age, the duration of symptoms, the size and location of tumor, histology, surgical strategy, and adjuvant therapy. Age and pathologic grade were admitted as the impact factors [5, 6]. All of the practicable therapy can be selected for ASS-LGG, but there is no enough evidence to prove which the criteria is. We analyzed the ASS-LGG patients’ data and compared their clinical characteristics in order to screen out the impact factors for functional status in ASS-LGG patients, which might provide some references for the patients’ surgical strategy.

Some authors have observed that age is a determinant for prognosis: the younger the patient is, the better the prognosis is [6–9]. In our research, we focused on the change of KPS before and after surgical intervention, analyze the amelioration of functional status which the patients gain through surgery, and found that the improvement of KPS through surgery was better in age less than 40.

Tumor less than 5 cm in diameter have more favorable prognosis than more than 5 cm. The volume of the tumor obviously increased the difficulty of surgery. However, even for those patients whose tumor sized less than 5 cm in diameter, the patients could not get significant improvement of functional status after surgery when their initial symptom was epilepsy.

It was a negative prognostic factor for the tumor in the left hemisphere [10, 11]. Because the left hemisphere is dominant sphere compared with right hemisphere and contains much connecting and projecting fibers, surgery might destroy the functional area and cause neurological deficits, which would absolutely decrease the patients’ functional status [7, 12]. The exact lobe of tumor distribution is not an impact factor [13]. We found that the patients with partial resection have better improvement of functional status if tumor just extrudes or partially infiltrates the functional area. But this did not cause difference in average improvement of KPS.

Surgery is the first option in the treatment of ASS-LGG until today [14]. But there is still controversy on whether extensive resection should be done [1, 2, 15–18]. In our study, for those whose tumor was located in the functional gyrus, the patients have better amelioration of KPS when a conservative resection was done. Therefore, a limited but safe resection is beneficial to confirm the histological diagnosis of ASS-LGG, relieve the burden of the tumor, and protect the function.

In our study, the duration of symptom was not the relative factor for improvement of functional status. Some potential risks existed in the delayed operation patients, such as the tumor developing into high grade, irreversible nerve injure, and intractable epilepsy [19–23]. But there have been no randomized trials to analyze the benefit of surgery in early stage and the patients’ status [24, 25]. The strategy of management is “waiting and observation” after a biopsy in many centers [25, 26].

Many centers are more likely to treat ASS-LGG with immediate postoperative radiation, especially for those whose surgery is partial resection or pathology is grade II [27, 28]. Early postoperative radiotherapy seemed to postpone the time of progression of low-grade glioma [29, 30], but we did not find evidence to prove its effectiveness in the improvement of functional status.

## Conclusions

This study was based on the postoperative functional status analysis, which was validated by postoperative KPS elevation. To ASS-LGG patients, predicting the functional status after surgery depends on the patients’ age, size, location of the tumor, and extent of resection. Relieving the size of the tumor and acquiring the pathological diagnosis should be recommended instead of the en bloc resection in the functional area.

## Abbreviations

ASS-LGG: Adult supratentorial superficial low-grade glioma
KPS: Karnofsky performance status
LGG: Low-grade glioma
